# Fungal stress responses from the view of transcriptome vectors

**DOI:** 10.15698/mic2026.11.889

**Published:** 2026-11-02

**Authors:** Károly Antal, István Pócsi, Tamás Emri

**Affiliations:** 1 Department of Zoology, Eszterházy Károly Catholic University, Eger, Hungary; 2 Department of Molecular Biotechnology and Microbiology, Faculty of Sciences and Technology, University of Debrecen, Debrecen, Hungary; 3 HUN-REN–UD Fungal Stress Biology Research Group, University of Debrecen, Debrecen, Hungary

**Keywords:** adaptation vector, *Aspergillus*, response vector, RNA sequencing, stress adaptation, stress response, transcriptomics

## Abstract

Transcriptomic approaches are widely used to study the stress biology of fungi or other (micro)organisms. When three or more transcriptomes are compared, considering their relative positions can clarify their relations. Here we represent transcriptomes as points in an N-dimensional space (transcriptome space), where dimensions measure the transcriptional intensities of genes. In this space stress responses correspond to vectors from the pretreatment transcriptome to the transcriptome altered by the treatment. This approach enables us to create simple definitions that let us focus on the data measured in the experiment. In addition, we show (1) the importance of kinetic studies in comparing stress responses, especially when examining responses to changes of different magnitudes; (2) how a reference transcriptome with genes expressed near the upper and lower ends of their transcriptional range constrains stress responses; (3) why the pre-treatment difference between transcriptomes makes the evaluation process difficult. We also show that comprehending the results of combinatorial stress response experiments is disproportionately challenging because of the cumulative nature of the problems mentioned above in these experiments. We propose that considering the relations of transcriptomes as points in the transcriptome space provides a view that complements the standard gene-centered analyses. Developing a model that describes the processes resulting in the observed relative position of transcriptomes can help reduce the risk of misinterpreting transcriptional changes at the level of individual genes or gene groups.

## INTRODUCTION

In mycology, stress is usually defined as an environmental condition that prevents the optimal performance and/or threatens survival of fungi 1. Stress is frequently detected as a reduction in viability, growth, conidium or meiospore production, increase in the lag phase of growth, or upregulation of various protection mechanisms (*e.g.* trehalose, glycerol, heat shock protein, or antioxidant enzyme production) initiated by environmental changes 1–4. Studying stress responses means that we study fungi “in action”: We are interested in how they respond to changes in their environment rather than how they work under optimal conditions. It allows us to explore how fungi sense their environment, how their regulatory networks function, and how they adapt to their ever-changing environment. The results can shed light on how pathogenic fungi survive in the body of the host organism, or how fungi occupy “artificial” habitats, *e.g.* a fermenter, stored food and feed, or art treasures 5–10. The knowledge gained can be applied to modulate fungal stress responses, either to promote or to inhibit their effective adaptation.

Transcriptomic approaches are widely used to investigate fungal stress responses. In most cases, transcriptomes of two culture types are compared, for example treated and untreated samples. Such analyses provide a comprehensive overview of transcriptional changes induced by a given treatment, enabling the identification of metabolic and signaling pathways involved in the stress response, as well as key genes and their regulators 11–13. A major advantage of this approach is that it does not require the examination of gene activity on an individual basis, as a single experiment yields information on all expressed genes. Consequently, transcriptomic analyses often reveal the importance of specific subprocesses that might remain undetected using non-omics approaches. These studies frequently serve as an initial step, helping to narrow the focus to a limited number of key genes or proteins. Then these genes or proteins can be used to modulate stress responses of fungi.

However, in some cases multiple treated (and untreated) cultures are analyzed, for example when comparing the effects of different stressors, varying stress levels, distinct culturing conditions, or when different strains are studied. In such experimental setups, the immediate goal is not necessarily to reduce the system to a small set of (potential target) genes or proteins. Instead, rather than adopting a strictly gene-centric perspective, the primary aim is to explore the global characteristics of the transcriptional response. For instance, stress responses often depend on the intensity of the stressor. In some cases, stronger stress results in a more pronounced regulation of the same set of genes observed under milder conditions. In other cases, however, fundamentally different gene sets may be regulated depending on stress severity 14, 15. This distinction is particularly relevant when selecting potential gene or protein targets to modulate stress responses. When comparing multiple stress conditions, it is also important to assess the extent of overlap between the corresponding transcriptional responses. A substantial overlap may indicate the presence of a general (environmental or core) stress response, consisting of a large stressor-independent component and a smaller stressor-specific component 16, 17. Genes upregulated as part of the general stress response are often associated with preparation for future stress events (cross-stress protection), rather than with coping with the immediate stress 16. Therefore, determining whether a potential target gene or protein belongs to the general stress response is of particular importance. Furthermore, in natural environments stressors typically act simultaneously or sequentially. Accordingly, transcriptomic studies are often designed to investigate how stress responses are influenced by additional stressors or, more broadly, by varying culturing conditions 18–21. Such insights are essential for identifying targets that remain effective in complex environments, such as the human host. In these types of studies, the objective is typically not to construct a detailed mechanistic model of a specific stress response under a single condition. Rather, the goal is to develop a more general - albeit less detailed and mechanistic - model describing adaptive strategies to changing environmental conditions.

Interpretation of transcriptome data is sometimes challenging. For example, the number of core stress response genes identified in a study highly depends on how strictly we define “uniform behavior”, or what kind of reference culture we apply 17, 22. Or, when we compare the stress response of *e.g.* a mutant and the reference strain, or the same strain under different culturing conditions, the difference between the transcriptomes of the strains before the stress treatment can influence their stress responses as much as the mutation or culturing condition does, and separation of the two effects can be difficult or even impossible 17, 21.

Here, transcriptomes are represented as points in an *N*-dimensional space (the transcriptome space), where each dimension corresponds to the transcriptional intensity of a gene. We discuss how the relative positions of transcriptomes reflects key aspects of stress responses that are relevant for the reliable interpretation of the observed changes.

## RESULTS AND DISCUSSION

### Theoretical background

#### The transcriptome space, assumptions, and terms used for states

To avoid ambiguity, we distinguish between three levels of the transcriptome. The biological transcriptome comprises the complete set of RNA molecules present in a cell under a given condition. The experimentally accessible transcriptome represents the subset of these molecules that is retained after RNA extraction and library preparation and is therefore determined by the experimental protocol. Finally, the computationally analyzed transcriptome comprises the transcripts that can be inferred from the sequencing data using the selected bioinformatic workflow. Its composition is therefore constrained by the experimental data as well as by the reference genome and annotation, or, alternatively, by the performance of transcript assembly methods. In the present study, the computationally analyzed transcriptomes comprised annotated protein-coding transcripts represented in the reference genomes of *Aspergillus fumigatus* Af293 and *Aspergillus nidulans* FGSC A4 (Supplementary Table 1). Accordingly, throughout this work the term transcriptome refers to this computationally defined set. However, the proposed conceptual framework is not inherently limited to protein-coding mRNAs and can be readily extended to other RNA classes, as well as to unannotated transcripts, provided that appropriate sequencing data are available.

We describe a transcriptome as a point in an *N-*dimensional space (transcriptome space) where the coordinates of the point represent the transcriptional activities of the genes. In a fixed environment, we expect the transcriptome to settle in a (more or less) fixed steady state. Upon a change to another fixed environment, we expect some transient changes of the transcriptome followed by settling in another steady state. We will use the terms “adapting” and “adapted” (relatively unchanging; “steady”) to refer to these two behaviors. To the extent a fixed environment uniquely determines the corresponding adapted state, this latter is independent of the history of the culture. Differences between such adapted states do not depend on the order these environments were presented to the culture. Note that the assumption that a fixed environment results in a steady state that is independent of the history of the culture relies on the effects of epigenetic regulation, stress response memory, transcriptional changes in cell cycle and clock genes on transcriptomes being negligible. Assuming that (1) the changes in the transcriptome are adaptive and (2) fitness is entirely determined by the environment and the transcriptome, we can think of the effect of the environment as prescribing a fitness landscape that guides the transcriptome towards its local maxima corresponding to the best achievable performance of the fungus under given biological constraints in that environment. Changing the environment changes the landscape and, in particular, the locations of the local maxima, therefore the fungus may have to modify (adapt) its transcriptome to re-optimize its fitness whether or not its fitness decreased (Fig. 1**A**).

In a typical stress biological experiment we have “unstressed cultures”, “stress treated cultures” (cultures under stress) and/or “stress-adapted cultures”. Researchers have proposed various definitions of stress, typically based on changes in one or more properties of the studied cultures (*e.g.*, alterations in growth, conidia production, accumulation of “protective” metabolites, or upregulation of “protective” enzymes) 1–4, 17, 23, 24. Based on these criteria, they implicitly define what constitutes stress-treated (stressed) and untreated (unstressed) cultures. As these approaches are not necessarily equivalent, they may lead to different interpretations of what constitutes “stress” across studies. To avoid such misunderstandings and to clarify the perspective adopted here, it is important to declare the definitions for the studied cultures used in this study. We regard the stress response as an adaptation to environmental changes, aimed at increasing fitness. We will use the following qualifiers: “control” (short for “control adapted”) for cultures adapted to the conditions applied before the treatment, “adapting” for cultures adapting to the environmental changes caused by the treatment, and “treatment adapted” for cultures that have adapted to the new environment. Adapting cultures have moving transcriptomes, while the control and treatment adapted cultures have steady transcriptomes (Figs. 1**A** and 1**B**). Besides the positions of the transcriptomes in the control and treatment adapted cultures, the (stress) response also includes the path between (Fig. 1**B**). Note, the “control” and “treatment adapted” labels are not intrinsic to these states, they simply denote the states adapted to the environments before and after the intervention. An experiment using the same environments in reverse order would swap the labels of the corresponding steady states. Still, the path taken between the two end points may depend on the order.

**Figure 1:**
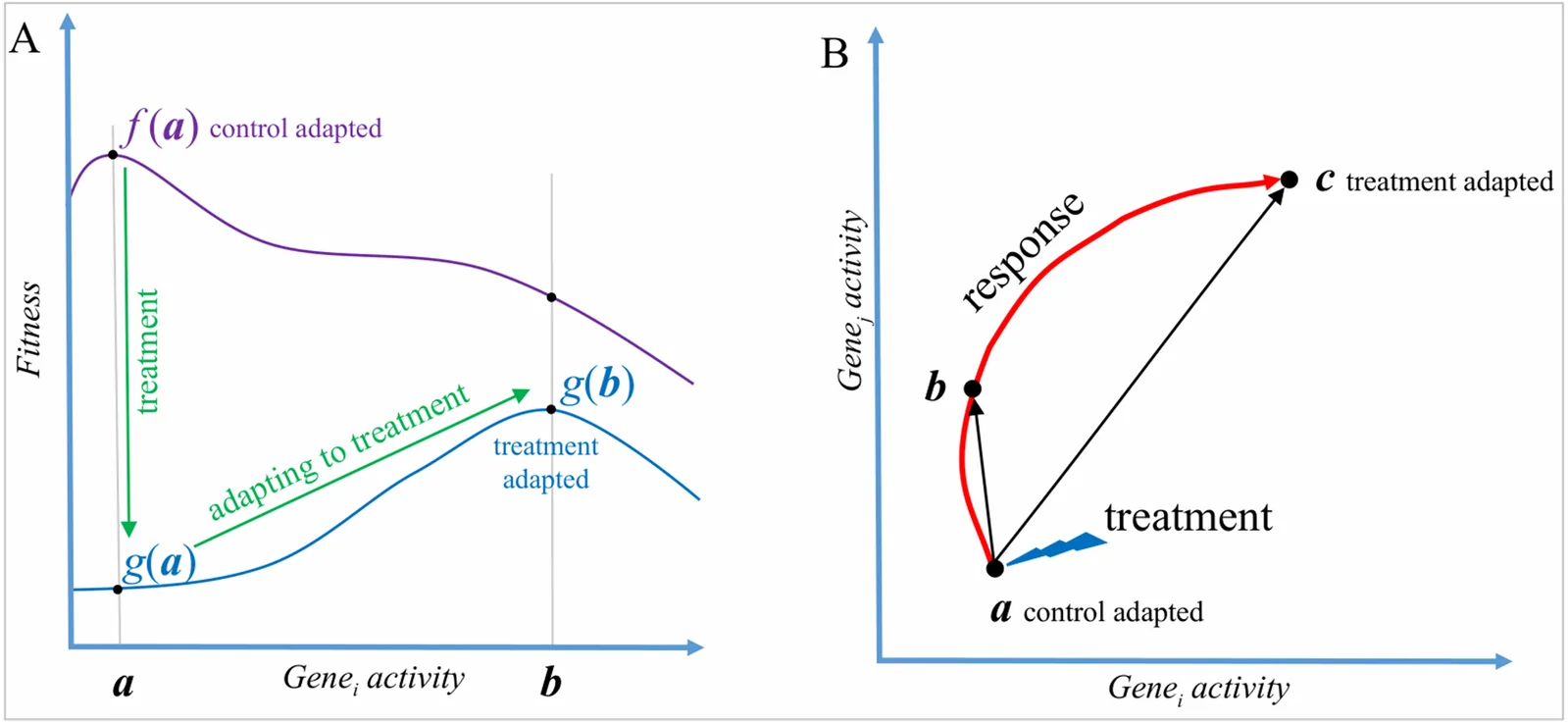
Responses in the transcriptome space. **(A)** shows the fitness landscapes *f* (purple) for the control environment and *g* (blue) for the treatment environment. Only one dimension of the transcriptomes is shown for clarity. In an idealistic situation when all transcriptional changes have adaptive value, the initial adaptation to the control environment moves the transcriptome to ***a***, with a control adapted maximal fitness *f*(***a***). Treatment changes the fitness to *g*(***a***), and adaptation increases it to *g*(***b***), corresponding to a treatment adapted culture. In this case, treatment reduced both the fitness and the available maximal fitness. Neither of these are necessary, unless we chose the control as the environment providing the highest maximal fitness: in general *g*(***b***) and even *g*(***a***) could be higher than *f*(***a***). Adaptation is driven by the shape of the treatment landscape, not by the reduction of fitness due to the treatment. **(B)** shows movements of a transcriptome in a transcriptome space (restricted to two dimensions for clarity), from the control adapted point ***a***, along the red line to the treatment adapted point ***c***, *via* an intermediate point ***b***. ***c-a*** is the adaptation vector and ***b-a*** is a response vector at some time before full adaptation.

#### Nonlinear transcriptome trajectories with distinct response and adaptation vectors

The response can be described as a time-dependent location in the transcriptome space. This consists of the path taken and the timing along the path (from ***a*** to **c***via* ***b*** on Fig. 1**B**). The response at a time *t* can be characterized by a “response vector” that points from the control transcriptome to the transcriptome at a time *t* (***r*** (*t*)= ***b–a*** on Fig. 1**B**). The difference between the initial (control) and final (treatment adapted) state is the “adaptation vector”, (***c–a*** on Fig. 1**B**).

Note, the movement of the transcriptome may be non-linear and of variable speed 25–27. The response vectors may differ from the adaptation vector in both magnitude and direction (Fig. 1**B**). The optimum of fitness landscapes are not necessarily sharp, they may form a relatively broad plateau allowing different cells to adapt to the same treatment with distinct transcriptomes. Moreover, it is also possible that different cells reach the same transcriptome while following different trajectories.

When two or more response vectors are studied, we can compare their lengths and directions. For cultures with different initial states, we can also ask whether the treatment increased or decreased their distance.

$\underline{\text{Response vector magnitude.}}$ The length (magnitude) of a response vector (| ***r*** (*t*)|) depends on the transcriptional changes of all considered genes and provides a measure of the magnitude of changes. If at the beginning of the response the transcriptome moves with little change of direction, then the length of the response vector approximates the length of the path taken. Since the adaptation vector is the difference between two steady transcriptomes, it does not depend on time and its length can characterize the amount of (net) changes in the transcriptome needed for adaptation (adaptation vector magnitude).

$\underline{\text{Difference in the nature of response vectors.}}$ The direction of a response vector depends on which genes show large transcriptional changes and characterizes the “nature” of the response vector. The angle between two response vectors provides a measure of the difference of their directions (regarded as “difference in the nature of responses”). Close to 0^∘^ angle indicates that the two treatments affected the genes in similar proportions. Close to 180^∘^ angle also indicates similar proportions, but opposite direction. A 90^∘^ angle indicates orthogonal directions. This includes non-overlapping gene sets (where the gene-wise products are all zeros), but also other cases, where the sum of positive and negative gene-wise products cancel, resulting in a zero scalar product of the two vectors.

$\underline{\text{Diverging and converging responses.}}$ For response or adaptation vectors starting from the same point, the distance between their end points can be better interpreted if we also consider their magnitudes and the angle between them (Fig. 2**A**). Conversely, the presence of very close end points can help in interpreting response or adaptation vectors of different magnitudes and directions (Fig. 2**B**).

In the forthcoming sections we show how this approach can help the evaluation and interpretation of transcriptome data.

**Figure 2:**
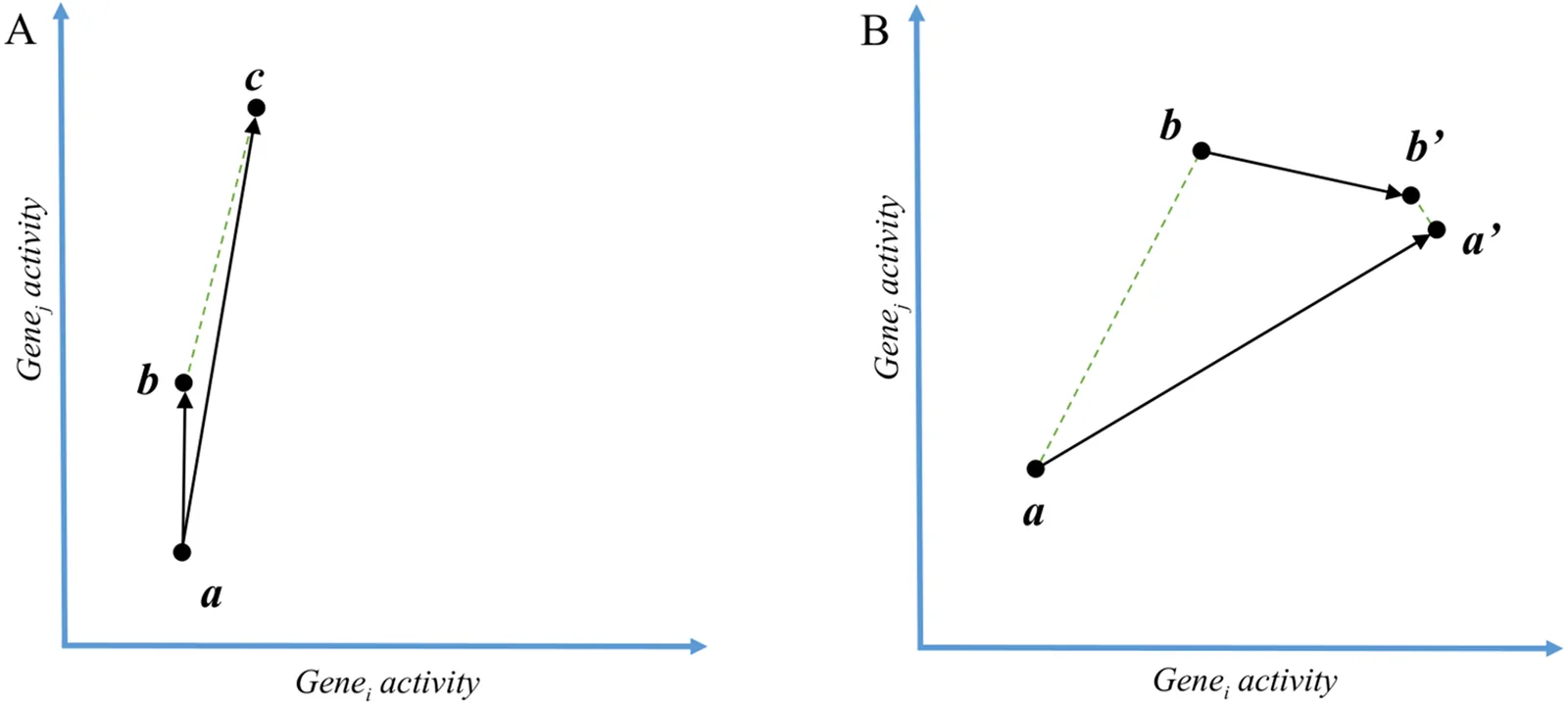
Features of transcriptome vectors. Only two dimensions of the transcriptomes are shown for clarity. **(A)** shows the transcriptome ***a***, adapted to control conditions, and the transcriptomes ***b*** and ***c*** adapted to different treatments. The dashed green line shows the distance between the treatment adapted cultures. Based on the distance between ***b*** and ***c***, the adapted transcriptomes differ. The small angle between ***b-a*** and ***c-a***, and the much smaller magnitude of the response ***b*-*a*** (**|b-a|**) suggest that this difference was primarily the consequence of the difference in magnitudes, not the different nature of the responses. **(B)** shows an experiment where the control ***a*** and ***b*** transcriptomes differ (*e.g.* because they belong to different strains or received different pretreatment). When treatment is applied, the transcriptomes change, and at some time later we observe the transcriptomes ***a’*** and ***b’***. Here we have responses with markedly different initial transcriptomes (dashed green line between ***a*** and ***b***), very different magnitudes (| ***a’-a*** | and | ***b’-b*** |) and nature (the angle between the ***a’-a*** and ***b’-b*** vectors is large). The very small difference between the two treated cultures (dashed green line between ***a’*** and ***b’***) adds an important detail: the transcriptomes seem to converge.

### Interpreting response magnitudes: apparent differences may reflect the same underlying behavior

Increased intensity of a treatment is likely to increase the resulting changes. This could be a proportional scaling up of the gene-wise amplitudes and/or speed of the response. In other cases we may find that a more intense treatment evokes a qualitatively different answer: some genes get disproportionally more or less involved, some having a discernible role only under increased or reduced treatment.

In Experiment 1 28 cultures of *A. fumigatus* Δ *meaB* gene deletion mutant (sensitive to the presence of NO^−^_2_) were treated with either 30 mM NaNO_2_ (low-NaNO_2_) or 90 mM NaNO_2_ (high-NaNO_2_) and early responses were recorded (30 min after treatment). According to the Venn-diagram of upregulated and downregulated differentially expressed genes (Fig. 3**A**), more genes responded to the high-NaNO_2_ treatment (2611 + 2017 = 4628 genes) than to the low-NaNO_2_ treatment (1286 + 586 = 1872 genes). Many of the genes responding to low-NaNO_2_ treatment also responded to high-NaNO_2_ treatment in the same direction (1094; 85% of the upregulated and 349; 60% of the downregulated genes). However, many genes showed upregulation or downregulation only under low-NaNO_2_ treatment (192 + 237 = 429 genes) or under high-NaNO_2_ treatment (1517 + 1668 = 3185 genes). These data may suggest that the two responses were similar (differing mainly in magnitude), but some concentration-dependent effects may also be relevant for both responses. Response vectors provide only a slightly different view of the changes (Fig. 3**B**). It also shows that the difference of the response vector magnitudes (blue and red arrows) can only partially ((148-84)/105 = 61%) account for the final difference (green line) between the resulting transcriptomes, the remaining part also depends on the difference in their directions, suggesting differential involvement of some genes. However, their contribution to the stress response appears to be smaller than in the Venn analysis (Fig. 3**C**).

Importantly, the differences between the two response vectors at identical delays do not imply different paths: while the two responses may follow distinct, non-overlapping response paths (blue and red dashed arrows), they may also both follow a path passing through both observed points (black dashed arrow) with different time courses. Consequently, we need kinetic studies to distinguish between the effects due to different paths and different time courses. The ambiguity between different paths and different time courses is particularly prominent when the response/adaptation vectors have different magnitudes. These can arise from different treatment intensities, different sensitivities of the strains compared, or different sensitivities due to different culturing conditions. Unfortunately, the kinetic studies necessary to resolve this ambiguity is often missing.

**Figure 3:**
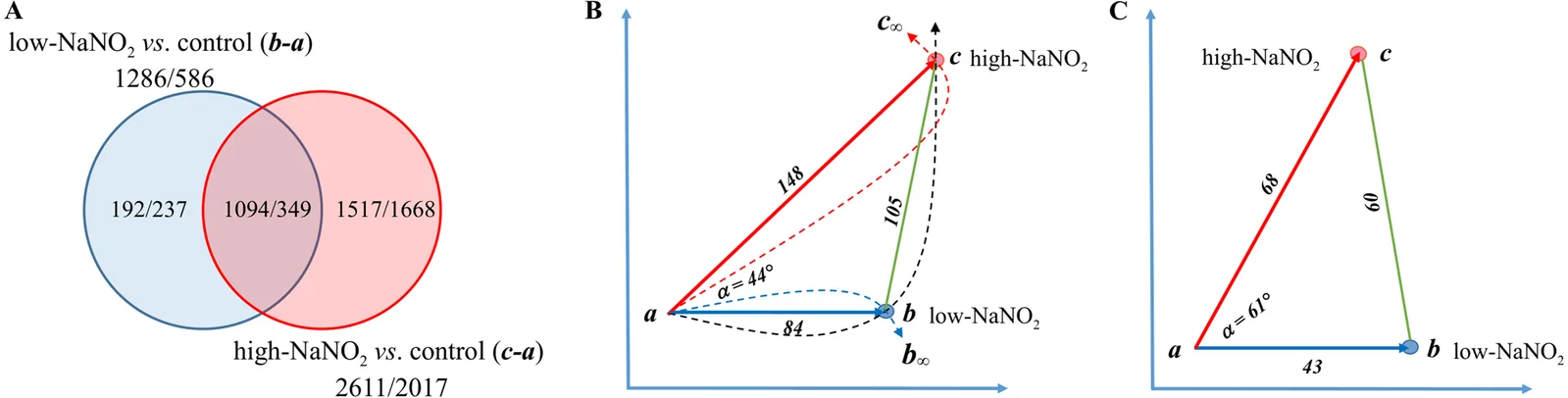
Comparison of the responses induced by low or high NaNO_2_concentration. (A) Venn-diagram of the upregulated/downregulated treatment responsive genes. The number of upregulated/downregulated genes under low-NaNO_2_treatment (left) and under high-NaNO_2_ treatment (right). These correspond to the ***b-a*** and ***c-a*** comparisons in Part B. Upregulated and downregulated genes were defined as differentially expressed genes (adj. *p* < 0.05) where |log_2_FoldChange| > 1. **(B)** Characterization of the two response vectors (***b-a***, blue arrow and ***c-a***, red arrow) corresponding to the same data as in part A by their lengths, their angle, and the distance between their end points (| ***c-b*** |, green line). The dashed blue and red arrows represent possible paths taken by the two stress responses, ending at the treatment adapted transcriptomes ***b***_***∞***_ and ***c***_***∞***_. The dashed black arrow represents an alternative explanation of the data: Both transcriptomes follow this path, but the changes in the transcriptome are faster after high-NaNO_2_ than low-NaNO_2_ treatment. Without kinetic studies we cannot decide which explanation is better. **(C)** Representation of the results summarized in the Venn diagram (Part A) by vectors for comparison. (Gene transcriptional activity was set uniformly to 0 in the control culture and to 1 (upregulated genes), −1 (downregulated genes) or 0 (non-responsive genes) in the treated cultures.) Note, α is 44^∘^ (part B) and 61^∘^ (part C). The ratio of the difference in response magnitudes and the final distance, (| ***c-a*** | - | ***b-a*** |) / | ***c-b*** |, is 61% (part B) and 42% (part C).

### The importance of reference transcriptome

#### Adapted cultures allow post hoc choice of reference

The co-regulated gene set (genes showing transcriptional changes in the same direction under each treatment) is frequently used to characterize transcriptome changes common among various stress treatments applied to control cultures. These common alterations are often regarded as general/core stress response 16, 17, 25.

Importantly, since (control or treatment) adapted cultures normally do not depend on the history of the culture, they can be compared in either order (Fig. 1**A**) or between treatments. When comparing the control to the treated cultures, the co-regulated gene set also characterizes how the fungus has adapted to the chosen control conditions 17, 22.

In Experiment 2, exponentially growing mycelia of *A. nidulans* were transferred into media containing glucose, lactose, arabinogalactan or no added carbon source and samples were taken after 12 h to allow cultures to adapt to the altered conditions 22. Selected properties of the adaptation vectors are summarized in Table 1 and Figs. 4**A** and 4**B**. Using cultures adapted to glucose as reference, the angles between the adaptation vectors were small (32^∘^ – 42^∘^) suggesting that adaptation to lactose, arabinogalactan or carbon starvation (*i.e.* adaptation to the absence of glucose) needed similar transcriptional changes. The number of co-upregulated and co-downregulated protein coding genes in these responses were 1192 and 917, respectively (with the “Venn-analysis”; defining treatment responsive genes as differentially expressed genes showing at least two times transcriptional changes) (Figs. 4**C** and 4**D**) 22. Since these are all adapted cultures, we can choose any of them as reference. Choosing lactose containing cultures as reference, the angles between the adaptation vectors were larger (49^∘^ – 89^∘^) and their lengths shorter (except for glucose). The observation that adaptation to either arabinogalactan or the absence of any carbon source is more similar to adaptation to lactose than to adaptation to glucose concurs well with the observation that arabinogalactan or the absence of any carbon source allow only slower, while glucose allows faster growth than lactose 22. Choosing lactose as control instead of glucose resulted in a co-upregulated and co-downregulated gene set of only 68 and 285 genes, respectively (Figs. 4**C** and 4**D**). Since the genes upregulated in the glucose vs. lactose comparison are downregulated in the lactose vs. glucose comparison, the co-upregulated (and also the co-downregulated) gene sets for the two reference cultures are disjoint.

**Table 1:** Characteristics of the responses in Experiment 2.

Comparisons			Length of the adaptation vector	Angles between the adaptation vectors^a^		
				Lac-Glu	Ara-Glu	C0-Glu
Lac	vs.	Glu	136	0^∘^	41^∘^	42^∘^
Ara	vs.	Glu	178	41^∘^	0^∘^	32^∘^
C0	vs.	Glu	177	42^∘^	32^∘^	0^∘^
						
				Glu-Lac	Ara-Lac	C0-Lac
Glu	vs.	Lac	136	0^∘^	89^∘^	88^∘^
Ara	vs.	Lac	118	89^∘^	0^∘^	49^∘^
C0	vs.	Lac	118	88^∘^	49^∘^	0^∘^

^a^ – The angles in the top block are measured between the three comparisons against glucose as indicated in the Comparisons column and at the top of the block. The bottom block shows angles between the comparisons against lactose. Abbreviations: Glu: glucose, Lac: lactose, Ara: arabinogalactan and C0: carbon starvation.

#### Extreme transcription levels constrain the direction of possible change

For each gene, its raw transcription level is limited by both its minimal and maximal possible values corresponding to finite limits for the coordinates in the transcriptome space. The corresponding point is thus restricted to a hyperrectangle (box) bounded by these limits. If the transcription of each gene is close to its minimum or maximum value, then the point is close to a corner of this box. From a corner position, each gene can only change in a single direction, and the angle between two possible moves of the transcriptome is at most 90^∘^. Thus, using a control transcriptome in a corner position precludes responses with opposing signs at the gene level and limits the angle between two possible responses at the transcriptome level to at most 90^∘^. When the control transcriptome is close to a corner, we can expect a similar, but less strict effect, still increasing the probability of responses with similar directions. In our example, we used the observed smallest and largest transcriptional activities of each gene as experimental limits instead of the unknown absolute limits. The transcriptome of cultures growing on glucose contained the most, while the transcriptome of cultures growing on lactose contained the fewest genes with the observed smallest or highest transcriptional activity (Table 2). Therefore, on glucose the movement of the transcriptome was more restricted by the borders of the transcriptome space than on lactose, and resulted in smaller angles between the adaptation vectors (Table 1) and a higher number of co-regulated genes. In other words, the fast growth on glucose is accompanied with strong downregulation of hundreds of genes and strong upregulation of other genes. Therefore, when any treatment preventing fast growth is applied, the transcriptional activities of these genes can only show large change in the opposite direction 17.

In summary, by studying transcriptome vectors we can obtain a more global view of the induced transcriptional changes that may complement the classic co-regulated gene set analysis (based on either Venn-diagrams or gene clustering 17, 25. Importantly, the corner position of the transcriptome chosen as reference can limit the possible changes (behavior of response/adaptation vectors), thus increasing the similarity between responses. This effect has to be taken into consideration during the design of the experiment and evaluation of the data.

**Table 2:** Characteristics of the four transcriptomes in Experiment 2.

Transcriptomes	Number of genes with		
	
	smallest rlog value	highest rlog value	$\$\Sigma^{a}\$$
Glucose	3972	3910	7882
Lactose	1749	1672	3421
Arabinogalactan	2035	2402	4437
Carbon starved	3111	2883	5994

^a^ – The sum of the “high” and “low” activity genes together. (The number of the studied genes was 10867.)

### Difference of initial transcriptomes may nontrivially affect responses

Many experiments compare transcriptomes from reference and mutant strains (with mutations affecting the abundance of a protein) cultured and treated identically. In order to understand the function of the protein encoded by the manipulated gene, we compare the response of the mutant and the reference strain to the treatment. We can directly compare the treated cultures (final difference), or we may attempt to eliminate the effect of the initial difference (between the transcriptomes of the control cultures) by comparing the response vectors of the two cultures. Unfortunately, these comparisons can be misleading without also taking into account the initial difference. Let us examine some simple cases for adaptation vectors (potentially based on partial transcriptomes) of a mutant and a reference strain. Let ***m*** and ***r*** denote the mutant and reference transcriptomes before the treatment (control transcriptomes for the mutant and the reference strains), ***m’*** and ***r’*** the transcriptomes after treatment (treatment adapted transcriptomes for the mutant and reference strains). In general, the four transcriptomes (***m***, ***r***, ***m’***, ***r’***) can be different and the two adaptation vectors are not necessarily in the same 2D plane (Fig. 5**A**). When the two initial or final transcriptomes, or the two responses are (almost) equal, then their relations become easier to interpret:

(1) “Divergence or convergence” case. If the mutation has negligible effect on the transcriptome under control conditions (***m*** ≈ ***r***) then the three different transcriptomes form a triangle. The adaptation vectors (***r’***–***r***) and (***m’***–***m***) ≈ (***m’***–***r***) may diverge (Fig. 5B) showing that the two strains adapted to the treatment with different transcriptomes. Similarly, the distance between the treatment adapted transcriptomes (***m’***–***r’***) can be close to zero (Fig. 5**C**; convergence). In this case, the two strains responded differently to the treatment even though they adapted to it with very similar transcriptomes.

**Figure 4:**
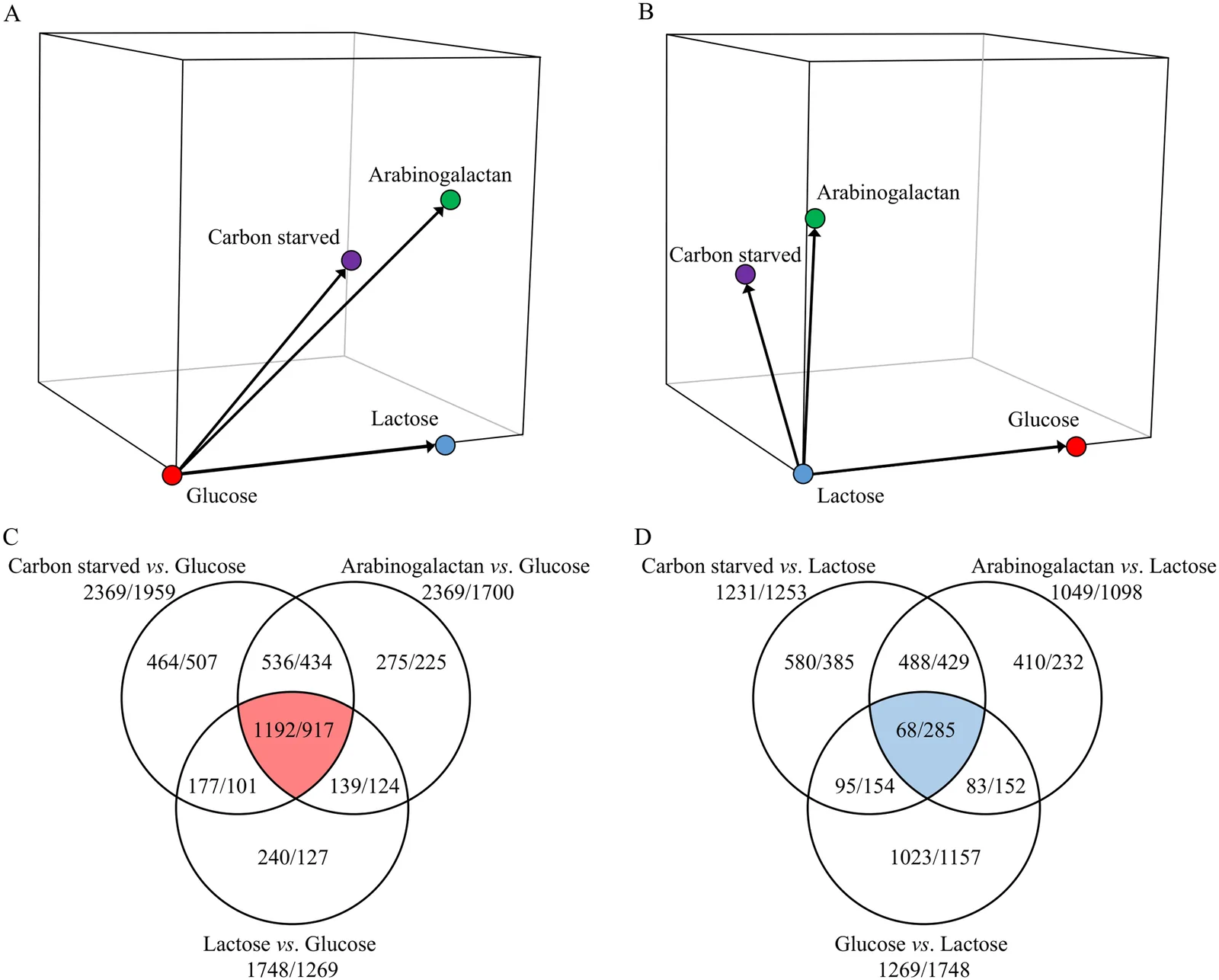
Adaptation vectors of *A. nidulans* cultures cultivated on glucose, lactose, arabinogalactan, or in the absence of any carbon source using cultures growing on glucose (A) or on lactose (B) as control (Experiment 2). For rotatable 3D view see Figs. S1A and S1B. The C and D panels show Venn-diagrams of the upregulated/downregulated carbon source dependent genes for comparison. Upregulated and downregulated genes were defined as differentially expressed genes (adj. p < 0.05) where |log2FoldChange| > 1.

**Figure 5:**
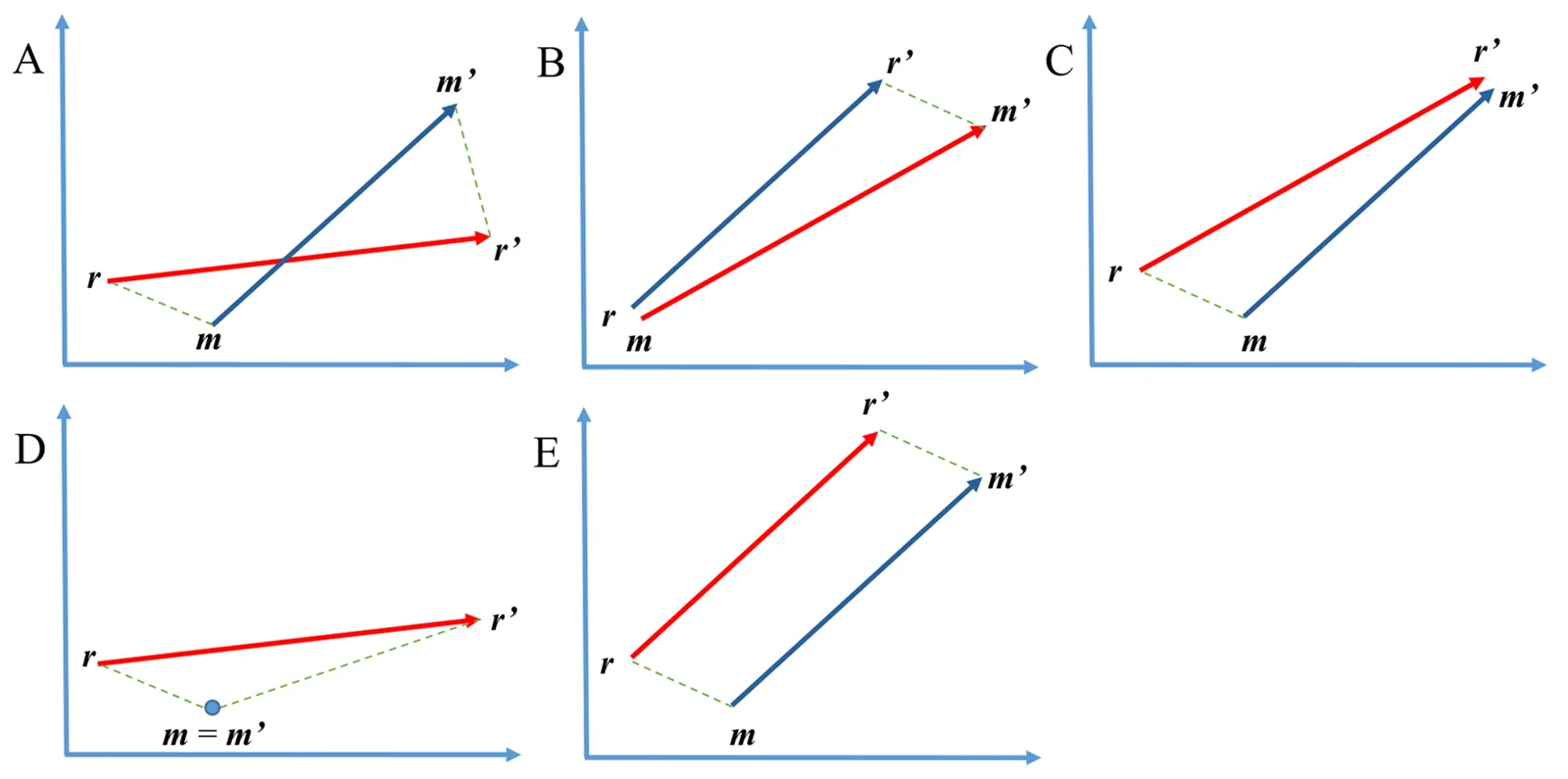
Possible response types of two different strains. Only two dimensions are shown. For clarity, vectors are similar in their length and close to the plane shown. Here **r** and **m** are the control cultures of reference and mutant strains, ***r’*** and ***m’*** the same cultures after treatment. The initial difference between the strains (***m-r***) compares the control cultures, the final difference (***m’***–***r’***) compares the cultures after treatment. The ***r’***–***r*** and ***m’***–***m*** differences are the adaptation vectors. **(A)** The directions of the two adaptation vectors are different ( α > 0^∘^). (Note, ***m’*** may be outside the plane of ***r***, ***m*** and ***r’***.) **(B)** Divergence. Almost identical transcriptomes before treatment: ***m***–***r*** ≈ 0. **(C)** Convergence. Almost identical transcriptomes after treatment: ***m’***–***r’*** ≈ 0. **(D)** Negligible effect in one strain. The treatment has no effect on the transcriptome in the mutant (***m*** = ***m’***). (Note, ***m***–***r*** can be zero.). **(E)** Parallelogram case. The two adaptation vectors are identical (***m’***–***m*** ≈ ***r’***–***r***).

(2) “Negligible effect in one strain” case. The control and treatment adapted transcriptomes can be close to each other for either of the strains (***m’*** ≈ ***m*** or ***r’*** ≈ ***r***) (Fig. 5**D**). This would demonstrate the critical role of the modified gene(s) either in the response in one strain (*e.g.* deletion of a gene prevents the development of the response) or in the prevention of the need to respond in the other (*e.g.* deletion of a gene enhance treatment sensitivity of the mutant leading to a response not observed in the wild type strain) (Fig. 5**D**).

(3) “Parallelogram” case. The effects of the mutation under control (***m***–***r***) and treatment (***m’***–***r’***) can be identical, or equivalently, the effects of the treatment on the mutant (***m’***–***m***) and reference strain (***r’***–***r***) can be identical (Fig. 5**E**). In these cases, the response appears to be independent of the mutation and equivalently the mutation appears to affect the transcriptome independently to the applied treatment.

Importantly, the ***m’***–***r’*** distance (“final distance between the strains”) depends not only on the ***r’***–***r*** and ***m’***–***m*** vectors but also on the ***m***–***r*** difference (“initial difference between the strains”). Therefore, evaluating only the adaptation (or response) vectors (comparing only the responses of the two strains) may lead to a different conclusion than evaluating the difference between the treatment adapted (or adapting) cultures.

In Experiment 3, *A. nidulans* and *A. fumigatus* cultures, a reference strain and a mitochondrial Mn-SOD gene deletion mutant ( Δ *sodB*) of each species, were treated with the superoxide generating agent MSB and the early responses were recorded (Fig. 6) 29, 30. In the case of *A. nidulans* (Fig. 6**A**), the lengths of the response vectors were similar (reference: 159, mutant: 183) and the response vectors were also close to parallel (24^∘^) (”Parallelogram” case; Fig. 5**E**), indicating that the effect of the missing gene on MSB-induced response was small. In line with this, the Venn-analysis resulted in a substantial overlap between the stress responsive gene sets recorded with the Δ *sodB* mutant and the reference strain (Fig. 6**C**; 29). In the case of *A. fumigatus* cultures (Fig. 6**B**), the directions of the two response vectors were different (65^∘^), the response length for the mutant (69) was larger than for the reference strain (30), and the treatment increased the distance between the strains (from 39 to 74) (“General” case; Fig. 5**A**). According to these data, the *sodB* gene deletion had a substantial effect on the response in this case. In line with this, the number of the differentially expressed genes showing at least twofold transcriptional difference between the two strains increased markedly by MSB treatment (Fig. 6**D**; 30) Note, in the case of *A. nidulans* comparing only the transcriptomes of the treated cultures (Fig. 6**C**; 29) may overestimate the importance of SodB in adaptation to the applied MSB elicited stress.

In Experiment 3, the behavior of partial transcriptomes for ribosome protein genes of *A. fumigatus* showed “divergence” (Figs. 5**B** and 7**A**): The initial distance between the strains was small (2.2) and increased *via* response vectors with very different direction (119^∘^) to more than twice after the treatment (5.6). These data indicate that the transcriptional response of ribosome protein genes were highly influenced by the lack of SodB. The difference between the partial transcriptomes of the control cultures may not influence substantially the outcome of the evaluation process in this case. In Experiment 4, early responses of *A. nidulans* to CdCl_2_ were studied using a reference strain and a mutant where the *atfA* gene (encoding a bZIP transcription factor 31) had been deleted 32. Fig. 7**B** shows the partial transcriptomes of the ribosome protein genes. In this case the initial distance between the strains (8.1) was reduced *via* response vectors with very similar direction (16^∘^) to less than half after the treatment (2.8) (”convergence”; Fig. 5**C**). In these experiments response vectors were studied (instead of adaptation vectors), therefore we have no information on possible further changes of the transcriptomes. Nevertheless, the data may indicate that in the presence of CdCl_2_, AtfA had minor effect of the transcription profile of ribosome protein genes, however under untreated conditions it had a substantial effect. Note, while the difference between the magnitudes shows that the two strains needed different “changes” in their partial transcriptomes (for ribosome protein genes) during adaptation to CdCl_2_, this was more due to the effect of the missing AtfA on their initial, and less on their final transcriptomes (Fig. 7**B**).

**Figure 6:**
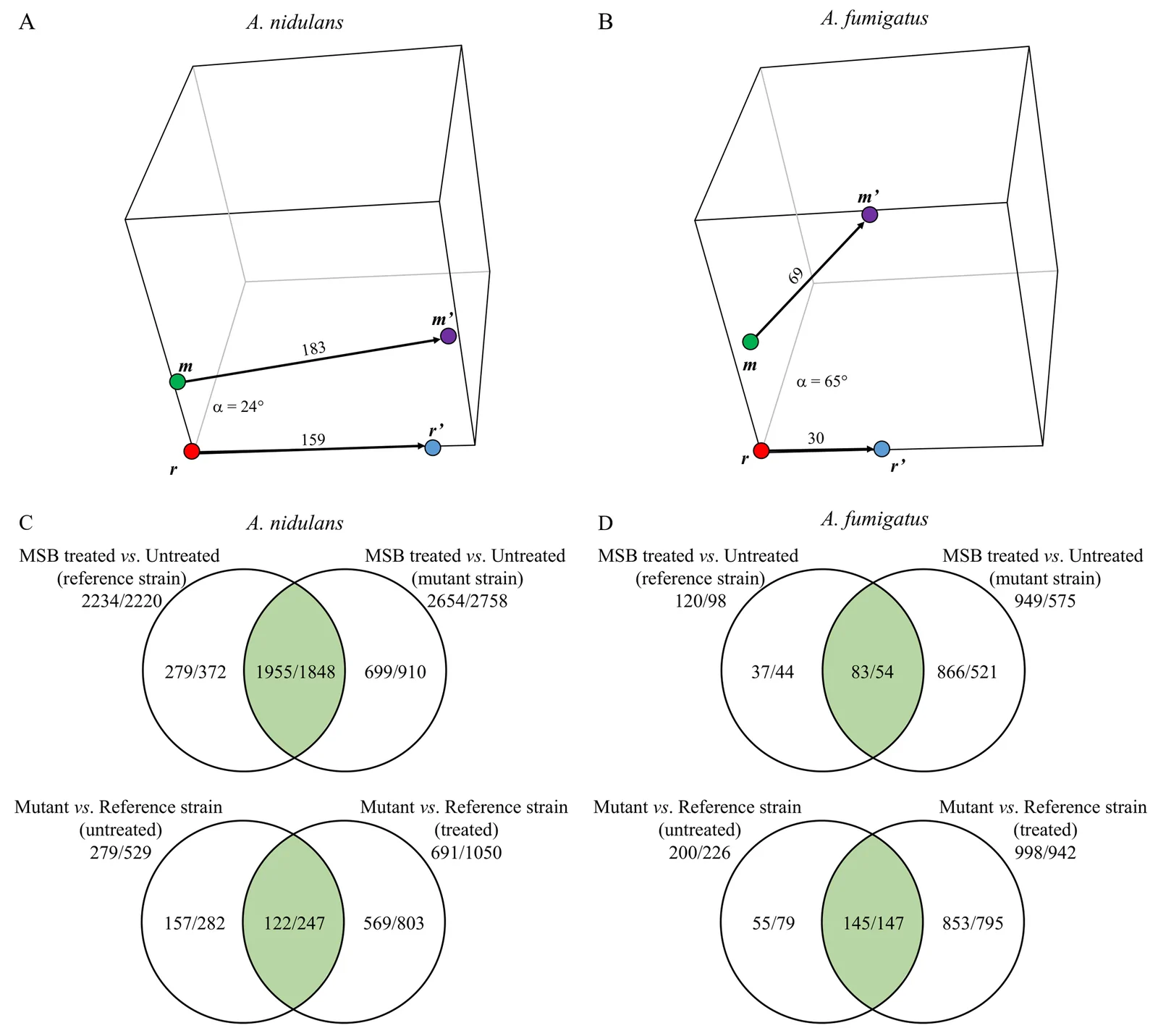
Response vectors of a mitochondrial Mn-SOD gene deletion mutant and a reference strain of *A. nidulans* (A) and *A. fumigatus* (B) (Experiment 3). The points represent the transcriptomes of control cultures of the reference strain (***r***), of the mutant strain (***m***) and the corresponding MSB treated cultures (***r’*** and ***m’***). On Panel A, ** |*m-r*|** = 59 and ** |*m’-r’*|** = 78, while on Panel B, ** |*m-r*|** = 39 and ** |*m’-r’*|** = 74. For rotatable 3D view see Figs. S1C and S1D. The C and D panels show Venn-diagrams of the upregulated/downregulated stress responsive and *sodB*-dependent genes for comparison. Upregulated and downregulated genes were defined as differentially expressed genes (adj. *p* < 0.05) where |log_2_FoldChange| > 1.

Mutation can alter either or both of the control and treatment adapted transcriptomes. Moreover, it may also be possible that the mutation only affects the path the transcriptome follows between the control and treatment adapted positions. All these effects contribute to the difference between the mutant and reference strain response vectors. During the evaluation of transcriptome changes, focusing on the response vectors or to the final between-strains difference and neglecting the initial between-strains difference can lead to misinterpretation of the data. When possible, minimizing the initial distance between the transcriptomes (or partial transcriptomes) of the strains by choosing appropriate culture conditions, or using *e.g.* optogenetic approaches to activate genetic modification simultaneously with the treatment – or applying a treatment that only affects one of the two strains – is a good strategy to reduce evaluation problems caused by the initial between-strains difference. However, if these approaches are not possible or reasonable, it is crucial to consider the initial distance in addition to the final distance and response vectors to minimize the possibility of misinterpreting the data.

**Figure 7:**
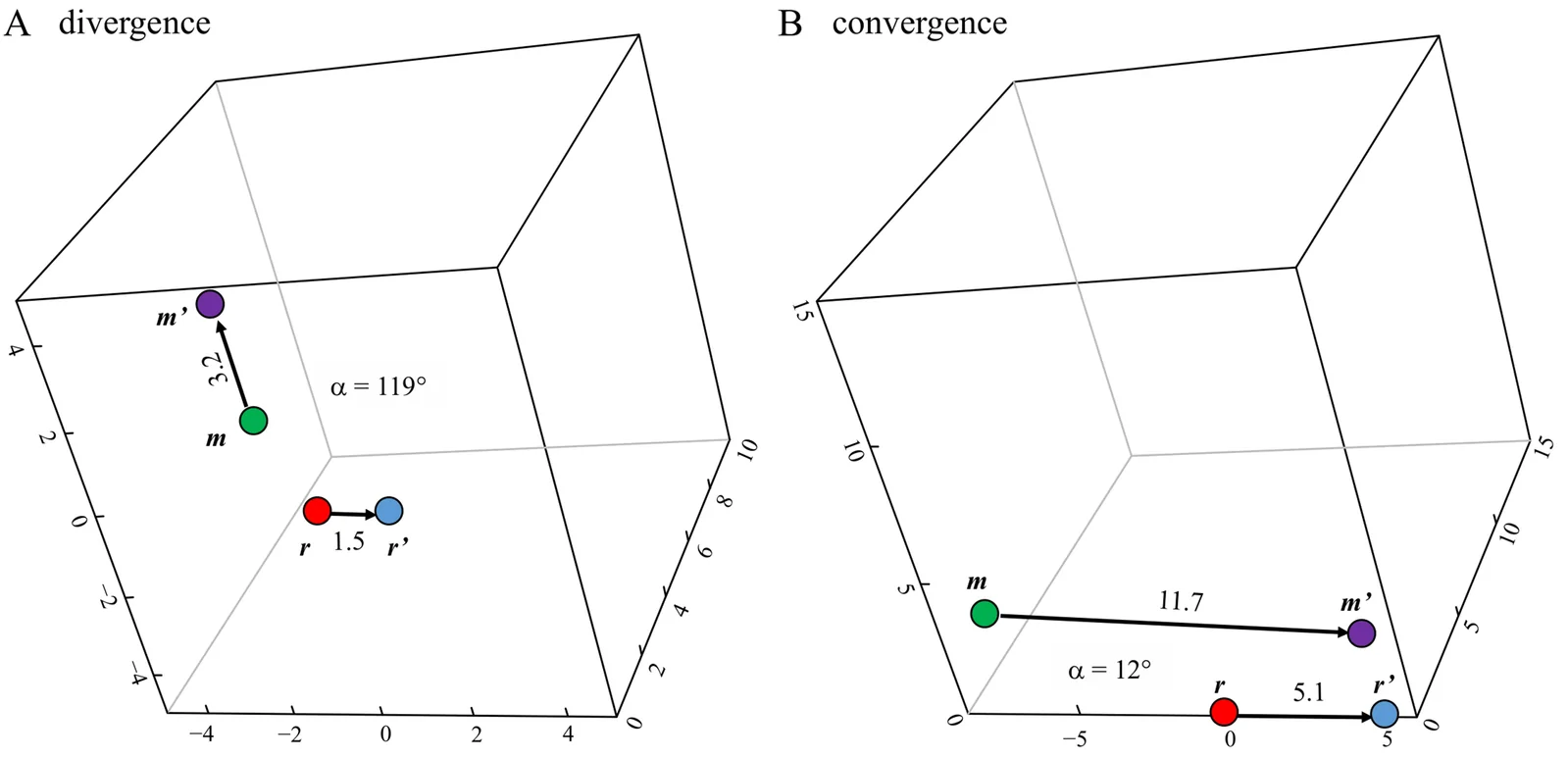
Response vectors calculated for ribosome protein genes. Examples for divergence and convergence. **(A)** The ***r*** and ***m*** points represent partial transcriptomes of control cultures of the reference and Δ *sodB* mutant *A. fumigatus* strains, while ***r’*** and ***m’*** are for the MSB treated cultures. | ***m-r*** | = 2.2 and | ***m’-r’*** | = 5.6. **(B)** The ***r*** and ***m*** points represent partial transcriptomes of control cultures of the reference and Δ *atfA* mutant *A. nidulans* strains, while ***r’*** and ***m’*** are for the CdCl_2_ treated cultures. | ***m-r*** | = 8.1 and | ***m’-r’*** | = 2.8. The ribosome protein genes were collected from the KEGG Pathway database (https://www.genome.jp/kegg-bin/show_pathway?ko03010). For rotatable 3D view see Figs. S1E and S1F.

#### Combinatorial stress amplifies challenges in interpreting changes in the transcriptome

In combinatorial stress experiments two (or more) different types of stressors are applied concomitantly (Fig. 8**A**) or sequentially on the same strain (Fig. 8**B**). These experiments revealed that the response to a treatment is highly dependent on culturing conditions or the pretreatment of the cultures 18, 21, 33, 34. Therefore, studying fungi under standard laboratory conditions may miss vital information on how fungi behave in the environment of our interest. It underlines the importance of *in situ* or *in vivo* studies (*i.e.* studying fungal stress responses in the nature, in animal models, etc.). Alternatively, we can investigate combinatorial stress responses (*i.e.* study the effect of a stressor under various conditions and/or in combination with various other stressors) to obtain a more global view on the behavior of the fungus. This approach can help to identify “mandatory” and “optional” elements of the response.

The problems of data evaluation are similar to those described in the chapters above. In fact, these problems are cumulative in this type of experiment. When stressors are applied concomitantly (Fig. 8**A**), we generally want to know if the response differs from the sum of the responses to separate applications of the individual stressors. Since for each gene the available range in the transcriptome space is limited, the response to a stressor of increasing strength necessarily saturates: unlimited linear scaling or linear summation of the response is not possible at the gene level. This also affects the summation of different responses affecting the same gene in the same direction. Moreover, a control condition placing a gene close to either limit of its available range only admits changes in the direction moving the gene away from this limit, thus induces similarities between the responses (“corner” effect). In sequential stress treatment experiments (Fig. 8**B**), we compare the responses to the same treatment applied on control and pretreated cultures. In this case, the difference between the transcriptomes of the control and the pretreated cultures (“pretreatment caused distance”) makes the evaluation process challenging. Moreover, when response vectors at a single point in time are studied, we cannot know whether we see two different responses or two different stages of the same response.

**Figure 8:**
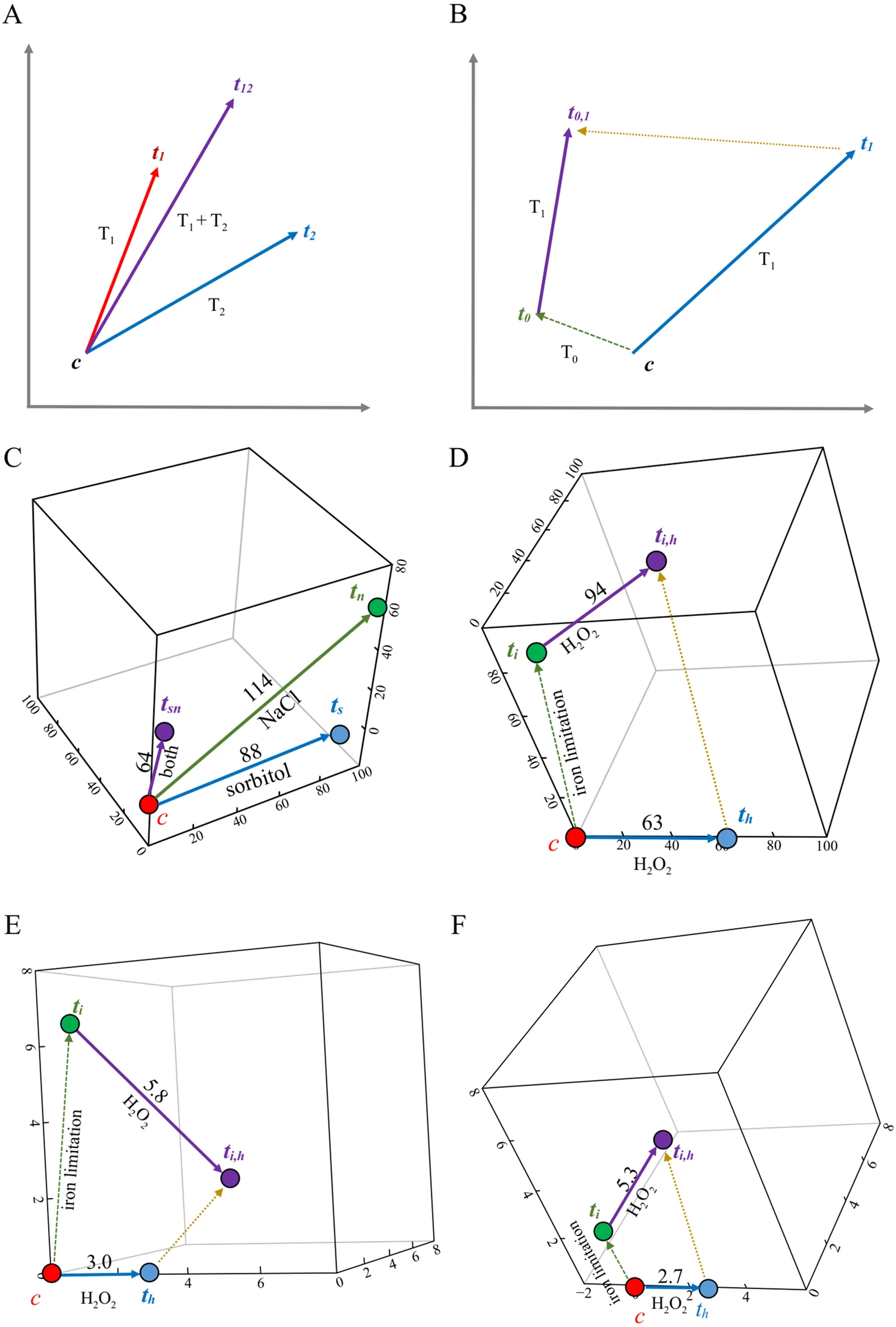
Stress vectors in combinatorial stress experiments. ** (A)** $t_{1}\;-$ ***c*** and ***t***_***2***_ − ***c*** are response/adaptation vectors induced by the T_1_ and T_2_ treatments, while ***t***_***12***_ − ***c*** is a vector induced by the concomitantly applied T_1_ and T_2_ treatments. ** (B)** $t_{0}-$ ***c*** is a response/adaptation vector induced by the T_0_ pretreatment. ***t***_***1***_ − ***c*** and ***t***_***0,1***_ − ***t***_***0***_ are response/adaptation vectors both induced by the same T_1_ treatment. ***t***_***0,1***_ − ***t***_***1***_ is the final difference between the transcriptomes. ** (C) *t***_***s***_ − ***c*** and ***t***_***n***_ − ***c*** are response vectors induced by sorbitol and NaCl treatments respectively in *A. fumigatus* cultures. ***t***_***sn***_ − ***c*** is the response vector after the combined sorbitol + NaCl treatment. The pairwise angles at ***c***: angle(***t***_***s***_ − ***c*** ***, t***_***n***_ − ***c***) = 32^∘^, angle(***t***_***sn***_ − ***c***, ***t***_***s***_ − ***c***) = 56^∘^, angle(***t***_***sn***_ − ***c***, ***t***_***n***_ − ***c***) = 71^∘^. ** (D–F) *t***_***i***_ − ***c*** is the adaptation vector induced by deferiprone (iron chelator) pretreatment in *A. fumigatus* cultures. The response vectors (***t***_***h***_ − ***c***) and (***t***_***i,h***_ − ***t***_***i***_) were induced by H_2_O_2_ in the absence or presence of deferiprone respectively. α (below) denotes the angle between these response vectors: α = angle(***t***_***i,h***_ − ***t***_***i***_, ***t***_***h***_ − ***c***). Panel D: The whole transcriptomes were studied. | ***t***_***i***_ − ***c*** |= 89, | ***t***_***i,h***_ − ***t***_***h***_| = 117, α = 85^∘^ Panel E: The partial transcriptomes for catalase-heme peroxidase genes were studied. | ***t***_***i***_ − ***c*** |= 6.6, | ***t***_***i,h***_ − ***t***_***h***_| = 3.3, α = 56^∘^. **(F)** The partial transcriptomes for thioredoxin-glutaredoxin-glutathione system genes were studied. | ***t***_***i***_ − ***c*** | = 2.3, | ***t***_***i,h***_ − ***t***_***h***_| = 6.5, α = 96^∘^. The composition of the catalase-heme peroxidase and thioredoxin-glutaredoxin-glutathione system gene groups is available in Emri et al. (2024). For rotatable 3D view see Figs. S1G, S1H, S1I and S1J.

In Experiment 5, the hyperosmotic stress response of *A. nidulans* cultures were studied after 2 M sorbitol, or 1 M NaCl, or 2 M sorbitol + 1 M NaCl treatments and the responses were recorded by RNA sequencing at 0.5 h after treatment 35. The direction of sorbitol and the NaCl elicited responses was similar (32^∘^) (Fig. 8**C**). However, the response to the combined treatment differed from those of single treatments both in magnitude (64 vs. 88 and 114) and direction (56^∘^ and 71^∘^) (Fig. 8**C**). This effect of the combined treatment was explained with an antagonistic interaction between sorbitol and Na^+^ 35–37. However, we cannot exclude that weaker single treatments (or sampling earlier in these cases) would show a more linear summation of the effects.

In Experiment 6, the H_2_O_2_ induced early responses of *A. fumigatus* cultures were studied by RNA sequencing using deferiprone iron chelator pretreated (iron-limited) and iron-supplemented (control) cultures 38. The lengths and directions of the two response vectors differed markedly (** |** $t_{h}\;-\;$ ***c*| **= 63, ** |** $t_{\mathrm{i,h}}\;-\;$ ***t***_***i***_**|**= 94, and α = angle(***t***_***i,h***_ − ***t***_***i***_, ***t***_***h***_ − ***c***) = 85^∘^; Fig. 8**D**) suggesting that responses were highly dependent on the pretreatment. For proper evaluation of the changes in the transcriptomes or partial transcriptomes for selected gene groups both the different response magnitudes and the substantial pretreatment caused difference should be taken into consideration. For example, in the case of partial transcriptomes for catalase-heme peroxidase genes, the two responses to H_2_O_2_ treatment had different directions ( α = 56^∘^; Fig. 8**E**). However, these different changes in the partial transcriptome reduced the difference (from 6.6 to 3.3) between the iron-supplemented and iron-limited cultures (convergence; Fig. 8**E**). In contrast, in the case of partial transcriptomes for thioredoxin-glutaredoxin-glutathione system genes, the very different responses to the same treatment ( α = 96^∘^) increased the pretreatment caused distance from 2.3 to 6.5 (divergence, Fig. 8**F**). Again, the different response magnitudes (Fig. 8**F**) raise the possibility of the same adapted partial transcriptome achieved with different speeds.

## CONCLUSION

In studying stress responses, the molecular biological approach – *i.e.*, the reduction of a complex system to a limited number of key proteins or genes to construct mechanistic models – is highly valuable, particularly when the aim is to manipulate or engineer such responses. However, an “omics” perspective may provide additional insight. Specifically, the “omics” perspective draws attention to genes exhibiting moderate or small transcriptional changes, since a gene’s contribution to fitness does not necessarily correlate with the magnitude of its transcriptional change. Moreover, the fitness cost of a response or adaptation may depend more on the large fraction of genes with moderate transcriptional changes than on a few genes with pronounced changes. In addition, this perspective emphasizes the integrative nature of the transcriptome. Gene regulation is not independent, as the contribution of a gene to fitness depends on the current cellular context in which its product operates – context that is assumed to be reflected by the current state of the transcriptome. Furthermore, the up- or downregulation of a given gene may be compensated for by changes in the expression of other genes, for example when the fitness landscape exhibits a relatively broad optimum.

To apply an omics perspective, transcriptomes were treated here as integrated systems and represented as points in an *N*-dimensional transcriptome space. Importantly, adopting such an omics perspective does not necessarily require specialized analytical methods; rather, it primarily involves a shift in conceptual framework.

Principal component analysis (PCA), which is routinely used in transcriptomic studies, is generally suitable for visualizing the relative relationships among transcriptomes. More detailed evaluation of distances and angles becomes necessary when the number and diversity of transcriptomic states are such that a two- or three-dimensional projection does not adequately capture the underlying high-dimensional structure. PCA typically enables rapid comparison of both the magnitude and the nature of transcriptomic changes, and can also be used to assess initial differences between reference transcriptomes when stress responses of different cultures or strains are studied. By distinguishing between steady and moving transcriptomes, it is possible to formulate alternative scenarios for how the global structure depends on the (unknown) trajectories of the latter. The “corner position” of a reference transcriptome (arising from extreme transcriptional values) can also be evaluated, for example by examining the rlog-transformed values, provided that a sufficiently diverse set of transcriptomes is available.

This perspective can guide the choice of an appropriate evaluation strategy, helping to avoid biases arising from corner positions of reference transcriptomes, their initial differences, or differences in the magnitude of transcriptomic changes. It may also facilitate the conceptualization of alternative biological scenarios underlying the data, prompting caution or motivating additional experiments when the unknown trajectories of moving transcriptomes remain ambiguous.

## MATERIALS AND METHODS

### Transcriptome data sets used in the study

All transcriptomic datasets included in this study were generated using poly(A)-enriched RNA libraries and single-end 75-bp sequencing. Sequencing reads were analyzed using a reference-based alignment workflow (Supplementary file 1). The transcriptomic datasets used in this study were generated in the following experiments, which are briefly summarized below:

Experiment 1: In this experiment, an *A. fumigatus* strain (VKmeaB1; Δ *meaB* gene deletion mutant) was grown in a minimal medium, and cultures in the exponential growth phase were treated with 30 mM NaNO_2_, 90 mM NaNO_2_, or kept untreated and samples were taken 30 min after treatment 28. Gene Expression Omnibus (GEO; http://www.ncbi.nlm.nih.gov/geo/) accession number: GSE277801.

Experiment 2: The *A. nidulans* THS30 reference strain was grown in complex medium and the mycelia were transferred into minimal media either containing no carbon source or a single carbon source (20 g/L glucose, 20 g/L lactose, or 20 g/L arabinogalactan) for 24 h 22. GEO accession number: GSE189016.

Experiment 3: The *A. nidulans* THS30 and *A. fumigatus* akuB^KU80^ reference strains as well as the *A. nidulans* and *A. fumigatus* mitochondrial Mn-SOD (SodB) gene deletion mutants ( Δ AN5577 and Δ Afu4g11580, respectively) were cultured in minimal medium until mid-exponential growth phase, then treated with 0.16 mM MSB (*A. nidulans*) or 6 mM MSB (*A. fumigatus*) for 30 min to achieve an approximately 30-40% growth reduction in the case of the reference strains 29, 30. GEO accession numbers: GSE244381 and GSE269970.

Experiment 4: The *A. nidulans* THS30 reference strain and the TNJ92 Δ *atfA* gene deletion mutant were cultured in minimal medium until mid-exponential growth phase, then treated with 0.2 mM CdCl_2_ for 30 min 32. GEO accession number: GSE166128.

Experiment 5: The hyperosmotic stress responses of the *A. nidulans* THS30 reference strain elicited by either 2 M sorbitol, or 1 M NaCl, or 2 M sorbitol + 1 M NaCl were compared 35. GEO accession number: GSE255841.

Experiment 6: The oxidative stress responses (elicited by 75 mM H_2_O_2_ and detected 1 h after treatment) of the *A. fumigatus* Af293 reference strain were compared under iron-limited conditions (in the presence of 1 mM deferiprone as iron chelator) and in the presence of sufficient amount of iron 38. GEO accession number: GSE256524.

### Evaluation of transcriptome data

Transcriptomes were represented as points ***a*** *=* (*a*_1_*, …, a*_N_), where *a*_i_ are the mean of rlog values for gene *i* produced by DEseq2 39 for the biological replicates. Here, *N* (the number of coordinates) is the number of genes studied (generally the number of genes occurring in the genome, or involved in a biological process of interest).

The difference between two points, ***a*** and ***b***, (observed under conditions A and B) is expressed by the vector from ***a*** to ***b***: ***c = b – a***, where ***c*** = (*c*_1_*, …, c*_N_), and *c*_i_ = $a_{i}-b_{i}$ . Note: since for large counts *a*_i_ and *b*_i_ are approximately on log_2_ scale, *c*_i_ approximates log_2_(FC_i_), where FC_i_ stands for the “fold change” value for gene *i*.

The length of this vector is the Euclidean distance between ***a*** and ***b***, $\left|c\right|=\sqrt{\sum c_{i}^{2}}=\sqrt{\sum {\left(b_{i}-a_{i}\right)}^{2}}$ and provides a measure of the overall amount of changes.

The angle α between two vectors (***a*** and ***b***) can be calculated from $\$\cos(\alpha )=\frac{\sum a_{i}b_{i}}{\left|a\right|\cdot \left|b\right|}\$$ .

To show the relative positions of four transcriptomes (***a***, ***b***, ***c***, ***d***) in a 3D plot, we used a Cartesian coordinate system with its origin at ***a***, the X axis pointing towards ***b***, the XY plane containing ***c***, and the 3D space also containing ***d*** (Supplementary file 2).

## SUPPLEMENTAL MATERIAL

All supplemental data for this article as well as rotatable 3D views for Supplemental Figures S1A-S1J are available online at http://www.microbialcell.com/researcharticles/2026a-antal-microbial-cell/.

Click here for supplemental data file.

## CONFLICT OF INTEREST

The authors declare no conflicts of interest.
