# Supplementary material for: Fungal stress responses from the view of transcriptome vectors
Source: Microb Cell. 2026 Nov 2;13:378–91. doi: 10.15698/mic2026.11.889 (PMC13756190; doi:10.15698/mic2026.11.889)
Supplement: Supplementary file 1 [file mic-13-378-s01.pdf]

**Supplementary file 1** Overview of the transcriptomic studies used

| ID | GEO[a] accession number | *Aspergillus* strain | Experiment | RNAseq[b] | Reference |
|---|---|---|---|---|---|
| Experiment 1 | GSE277801 | *A. fumigatus* Af293 and Δ*meaB* | Nitrosative stress response (induced by nitrite) was studied in submerged cultures. | Samples were collected in three biological replicates. Library preparation was performed using poly(A)-selected RNA. Single-read 75 bp Illumina sequencing was performed in one lane of a sequencing flow. The hisat2 software was used for mapping the raw reads and DESeq2 software was used to determine differentially expressed genes and calculate rlog values. | [28] |
| Experiment 2 | GSE189016 | *A. nidulans* THS30 | Carbon starvation and limitation stress responses were studied in submerged cultures on glucose, lactose, arabinogalactan or without any carbon source. | | [22] |
| Experiment 3 | GSE244381<br>GSE269970 | *A. nidulans* THS30 and Δ*sodB*;<br>*A. fumigatus* $akuB^{KU80}$ and Δ*sodB* | Oxidative stress response (induced by menadione sodium bisulfite) was studied in submerged cultures. | | [29-30] |
| Experiment 4 | GSE166128 | *A. nidulans* THS30 and Δ*atfA* | Heavy metal stress response (induced by $CdCl_2$) was studied in submerged culture. | | [32] |
| Experiment 5 | GSE255841 | *A. nidulans* THS30 and Δ*gfdB*;<br>*A. wentii* CBS141173 and An-*gfdB* | Hyperosmotic stress responses (induced by NaCl, sorbitol, or NaCl and sorbitol) were studied in submerged cultures. | | [35] |
| Experiment 6 | GSE256524 | *A. fumigatus* Af293 | Oxidative stress response (induced by $H_2O_2$) was studied in the presence and absence of glucose and/or iron in submerged cultures. | | [38] |

[a] - GEO: Gene Expression Omnibus (http://www.ncbi.nlm.nih.gov/geo/)

[b] – Further details are available in the shown reference and at the GEO accession number.

**Supplementary file 2** Coordinates used for plotting four points in three dimensions.

To show the position of four transcriptomes (***a***, ***b***, ***c***, ***d***) in a 3D plot coordinates of the four points (***A***, ***B***, ***C***, ***D***) were calculated as follows. While keeping the distances between the points, we moved ***a*** to ***A*** = (0,0,0), then rotated ***b*** to fall on the X axis,

***B*** = ($B_x$, $B_y$, $B_z$) = ($|b-a|, 0{,}0$), and ***c*** on the XY plane,

***C*** = ($C_x$, $C_y$, $C_z$) = $\left(\frac{|c-b|^2-|c-a|^2-|b-a|^2}{-2\cdot|b-a|}, \sqrt{|c-a|^2-C_x^2}, 0\right)$.

After these transformations ***d*** becomes $D = \left(D_x, D_y, D_z\right)$, $D =$

$$\left(\frac{|d-b|^2-|d-a|^2-|b-a|^2}{-2\cdot|b-a|}, \frac{|d-c|^2-|d-a|^2-C_x^2-C_y^2+2D_xD_y}{-2\cdot C_y}, \sqrt{|d-a|^2-D_x^2-D_y^2}\right)$$
